# Decreased Amygdalar Activation to NSSI-Stimuli in People Who Engage in NSSI: A Neuroimaging Pilot Study

**DOI:** 10.3389/fpsyt.2020.00238

**Published:** 2020-04-02

**Authors:** Jill M. Hooley, Mary Kathryn Dahlgren, Stephanie G. Best, Atilla Gonenc, Staci A. Gruber

**Affiliations:** ^1^ Department of Psychology, Harvard University, Cambridge, MA, United States; ^2^ Cognitive and Clinical Neuroimaging Core, McLean Imaging Center, McLean Hospital, Belmont, MA, United States; ^3^ Department of Psychiatry, Harvard Medical School, Boston, MA, United States; ^4^ McLean Hospital, Boston, MA, United States

**Keywords:** nonsuicidal self-injury, self-harm, functional magnetic resonance imaging, emotional processing, amygdala

## Abstract

In healthy individuals, stimuli associated with injury (such as those depicting blood or wounds) tend to evoke negative responses on both self-report and psychophysiological measures. Such an instinctive aversion makes sense from an evolutionary perspective. However, to engage in nonsuicidal self-injury (NSSI), this natural barrier must be overcome. The Benefits and Barriers model of NSSI predicts that people who engage in NSSI will show diminished aversion to NSSI-related stimuli compared to controls who do not engage in NSSI. We tested this hypothesis in a pilot study assessing 30 adults, 15 of whom reported current skin cutting and 15 of whom had no history of NSSI. Functional magnetic resonance imaging (fMRI) data were collected while participants viewed neutral, positive, and negative images selected from the International Affective Picture System. Participants also viewed NSSI images depicting razors, scalpels, or wounds caused by cutting. Compared to healthy control (HC) participants, the NSSI group showed decreased amygdala and increased cingulate cortex (CC) and orbitofrontal cortex (OFC) activation to NSSI and negative images. They also showed increased amygdalar and OFC activation to positive images. Neither the control group nor the NSSI group demonstrated significant activation within regions more typically associated with reward during any of the conditions; however, positive and negative affect ratings collected throughout the course of the task suggested that none of the affective conditions were viewed as rewarding. Although preliminary, these findings are suggestive of reduced limbic and greater cortical processing of NSSI stimuli in those with a history of this behavior. This has potentially important implications for current models of NSSI as well as for its treatment.

## Introduction

Most people have an aversion to the sight of blood and wounds (1, 2). The same is true for objects that threaten physical integrity such as knives, razors, scalpels, and pieces of broken glass. However, to engage in nonsuicidal self-injury (NSSI), this natural aversion must be overcome. NSSI involves deliberate self-inflicted injury to body tissue in the absence of any clear wish to die (3). A common form of NSSI is skin cutting (4). Knives, razors, glass, and other sharp objects are often used for this purpose.

The Benefits and Barriers model of NSSI developed by Hooley and Franklin (5) proposes that engaging in NSSI provides benefits. A major benefit is improved mood. NSSI tends to be used as an emotion regulation strategy (6, 7) and people who engage in NSSI report that it makes them feel better. Notably, NSSI is associated with a reduction in negative mood and an increase in positive mood (8, 9). In other words, both positive and negative reinforcements appear to play a role. Mood improvement can occur while experiencing pain [see (10, 11)] or following the termination of pain [pain offset relief; [see (10, 12)].

The Benefits and Barriers model also highlights several barriers to engagement in NSSI. A key barrier is aversion to NSSI type stimuli. As noted above, to engage in NSSI, any aversion to the sight of blood, wounds, razor blades, or similar must be overcome. The greater the aversion is, the less likely the behavior is to begin or to become instantiated. Correspondingly, a reduction in the instinctive avoidance of these stimuli has the potential to increase risk of NSSI engagement.

As NSSI is followed by emotional relief or mood improvement, stimuli such as knives or razors that are used in self injury are likely, over time, to become associated with well-being. This would be expected to occur in an automatic manner *via* the process of classical conditioning and would not require any formal or explicit learning. Repeated exposure to NSSI stimuli might also be expected to lead to diminished aversion to these stimuli over time through the simple process of habituation. Consistent with these ideas, research suggests that people who engage in NSSI report finding self-injury related stimuli less aversive than people who do not engage in NSSI. This is true for both explicit (13) as well as implicit measures (14, 15). Moreover, the greater their lifetime engagement in NSSI, the less aversive participants rate NSSI stimuli as being (14).

Two studies have also shown that diminished aversion to NSSI stimuli predicts future NSSI frequency in the near term (16, 17). Moreover, compared to controls who do not engage in NSSI, people who engage in NSSI demonstrate fewer difficulties inhibiting their behavior on a stop signal task in the context of exposure to NSSI stimuli (18). Indeed, relative to controls, their ability to halt the execution of an already intended action was enhanced after viewing a NSSI-related image, even though their overall performance (i.e., after viewing other types of emotional images) was worse. This again supports the idea that people who engage in NSSI are processing NSSI images in a manner that is different in some way and that they may be less perturbed by such images relative to controls.

Much remains to be learned about the neurobiology of NSSI behavior. Neuroimaging studies are often conducted using participants diagnosed with borderline personality disorder, making it difficult to isolate factors that may be specific to NSSI in other contexts. Given the brain changes that occur during adolescence, it is also likely that findings from samples of youth engaging in NSSI may yield different results from studies involving adult samples. However, there is some evidence that during emotional, social, and reward processing, individuals who engage in NSSI behaviors exhibit enhanced activation in frontal regions, including the cingulate cortex (CC), orbitofrontal cortex (OFC), and additional regions within the prefrontal cortex (19–23) as well as increased CC activation during a task of cognitive control (24). Anomalies in amygdalar circuitry have also been identified in female adolescents with a history of NSSI (25). In addition, there is preliminary evidence that pain—either caused by a thermal (heat) stimulus or from creating an experimenter-induced incision wound—may decrease amygdala activation and also normalize functional connectivity within key frontal areas (26, 27). This is consistent with the idea that NSSI may help regulate arousal and relieve stress in these individuals.

In the current pilot investigation, we used a region of interest approach to examine patterns of brain activation in people who engage in NSSI and in control participants who do not during exposure to a range of affective images. Given that studies have demonstrated altered activation in several ROIs in those who engage in NSSI behavior, we planned to examine three ROIs critical for processing emotion: the amygdala, CC, and OFC. Additionally, as there is evidence that NSSI is associated with improvement of mood in those who engage in this behavior, we planned to examine two ROIs typically associated with reward processing: the nucleus accumbens (NAcc) and ventral tegmental area (VTA). Images were drawn from the International Affective Picture System, (IAPS; 28). Some images depicted neutral scenes; others were more positive or more negative in nature. Importantly, we also included NSSI related images that depicted razors, scalpels, wounds, and blood.

We hypothesized that exposure to NSSI stimuli would be associated with lower levels of activation within the amygdala in participants who engage in NSSI behaviors compared to HCs. This prediction was made based on the idea that increased familiarity with NSSI would reduce aversion to NSSI related stimuli (5, 14) as well as on Reitz et al.'s findings (27) linking incisions (and perhaps therefore also images of incisions) to decreased amygdala activity. We also predicted increased activation in both CC and OFC during exposure to NSSI images. Increased CC activation during emotion processing has been noted in BPD patients (29) and in adolescent patients who engage in NSSI (22). The OFC is implicated in the subjective valuation of rewards and is considered to be a key region for the integration of sensory, hedonic and emotional information (30). Vega and colleagues (23) have also reported enhanced activation of the OFC in the context of reward in BPD patients with NSSI but not in BPD patients without NSSI. Our inclusion of other reward processing areas (NAcc and VTA) was more exploratory. Although NSSI is followed by affective benefits (5), people who engage in NSSI do not classify NSSI images as explicitly positive stimuli (18). Poon and colleagues (31) also found no association between thoughts of NSSI and altered reward processing in NAcc in adolescents. Therefore, we did not have any directional hypotheses regarding fMRI activation during exposure to NSSI stimuli in the NAcc and VTA.

## Materials and Methods

### Participants

Thirty community residents aged between 18 and 31 years of age (*M*=22.03, *SD*=3.51) were recruited from the Greater Boston area by means of online and posted advertisements. Only right-handed female participants were recruited in order to maximize homogeneity of the sample and because our NSSI images involved skin-cutting, which is more prevalent in females (32). Fifteen participants reported current engagement in NSSI by means of skin-cutting (≥10 lifetime episodes). The remaining 15 women were healthy control (HC) participants with no history of NSSI and no current psychiatric diagnosis. All participants completed the Wechsler Abbreviated Scale of Intelligence [WASI: (33)] to ensure that the groups were comparable with respect to general intelligence. Participants for this study are the same as those reported in Dahlgren et al. (24).

Exclusion criteria included head injury with loss of consciousness (≥10 min); any history of medical illness affecting cognition; neurological disorders; being a nonnative English speaker (required for the assessments), as well as MRI-related contraindications (e.g., metal implants, claustrophobia). From a total of 20 potentially eligible controls and 17 potentially eligible NSSI participants, 7 participants were excluded as they failed to respond to scheduling calls (*n*=3), declined to participate (*n*=1) or reported significant marijuana use during the study visit (*n*=3) and were therefore ineligible. Prior to participation, all study procedures were fully explained and participants provided signed informed consent in accordance with the Declaration of Helsinki. The study was approved by the Harvard University Committee on the Use of Human Subjects and the McLean Hospital Institutional Review Board.

### Diagnostic Assessments

Diagnostic information was obtained from all participants using the Structured Clinical Interview for DSM-IV Disorders I (34) & II (35). Control participants were excluded if they met criteria for any current diagnosis. Within the NSSI group, the most prevalent DSM disorders were borderline personality disorder (n=13; 86.67%), mood disorders (n=12; 80.00%) and anxiety disorders (n=8; 53.33%). One participant met criteria for an eating disorder (6.67%), and one participant met criteria for past alcohol dependence (6.67%). A history of at least one suicide attempt was reported by 4 of the NSSI participants (26.67%).

NSSI participants were also interviewed using the NSSI section of the Self-Injurious Thoughts and Behaviors Interview [SITBI 2.1; (36)]. The SITBI is a structured clinical interview that assesses the presence, age of onset, frequency, and other characteristics of self-injurious thoughts and behaviors. The SITBI shows strong interrater reliability (average κ =.99), high test-retest reliability over a six-month period (average κ =.70), and good concurrent validity as demonstrated by strong associations between the SITBI and other measures of NSSI [average κ =.87; see (36)]. All participants completed a battery of clinical rating scales to assess mood, emotional reactivity, and impulsivity.

### Clinical State Assessments

Clinical state and mood were evaluated using several standard self-report measurements. The State-Trait Anxiety Inventory [STAI; (37)] measures current anxiety levels (state) and general anxiety level (trait). The Beck Depression Inventory [BDI-2; (38)] provides a rating of overall depression. The Mood and Anxiety Symptoms Questionnaire [MASQ; (39)] reflects general distress from depression and anxiety-based symptoms and provides assessment of anxious arousal and anhedonic depression. The Positive and Negative Affect Schedule [PANAS; (40)] assesses positive affect associated with pleasurable engagement and negative affect associated with arousing aversive states. The Profile of Mood States [POMS; (41)] measures current mood state for the individual domains of vigor, anger, confusion, tension, and depression, and yields a composite measure of total mood disturbance.

Additionally, all participants completed two self-report measures of emotion regulation. The White Bear Suppression Inventory [WBSI; (42)] measures thought suppression, which is related to obsessive thinking and negative affect. The Emotion Reactivity Scale [ERS; (43)] assesses how emotions are experienced at the levels of sensitivity, arousal/intensity, and persistence.

Impulsivity was assessed using the UPPS Impulsive Behavior Scale [UPPS; (44)], a self-report measure of impulsivity comprised of four subscales: lack of premeditation, lack of perseverance, urgency (both negative and positive), and sensation seeking. Finally, as noted earlier, all participants completed the WASI (33), a measure of general intelligence (IQ).

### Affective Picture Task

Participants viewed a total of 48 stimulus images. The stimulus set consisted of 12 of the following picture types: neutral, positive, negative (non-NSSI), and NSSI. The positive, negative, and neutral images were selected from the IAPS (28), and were matched for arousal based on normative ratings. Across valence type (positive, negative, and neutral), images were selected that had average normative arousal ratings within the “not arousing and not unarousing” range. The NSSI picture set was developed by the first and third author. Five of the NSSI images depicted an individual pressing into her wrist a tool commonly used for NSSI (e.g., a razor, scissors, knife, etc.). The other five images showed a bleeding arm following cutting. These images varied in the number and severity of cuts, as well as in the resulting quantity of blood shown. They were obtained through an online Google image search for terms such as “NSSI,” “cutting,” and “self-injury.” Each picture's owner granted permission for her picture to be used in the study.

All images were presented to participants during an fMRI paradigm consisting of four affective conditions (subtests) completed in the following order: neutral, NSSI, negative, and positive. The total run time of each subtest was 2 min and 30 s and was comprised of 30 s fixation blocks (F), in which participants viewed a static, plus (+) sign on the screen, interleaved with 30 s stimuli presentation blocks (S) and presented in the following order: F,S,F,S,F. During each 30 s stimuli presentation block, six images were presented to participants for 4.5 s with a fixed 0.5 s interstimulus interval for a total of 12 images presented during each subtest. Images were presented randomly without replacement. To ensure that participants were actively engaged in the task, they were instructed to press a button as quickly as possible after each new image appeared on the screen; data on response time (ms) and omission errors were recorded. Participants also completed the PANAS immediately before the task as well as after each affective condition subtest to assess mood state changes occurring over the course of the task.

### Statistical Methods and Analyses

Univariate analyses of variance (ANOVAs) were used to compare the two groups on demographic and clinical variables. Two-tailed analyses were used to compare the demographic data, but since the NSSI group was expected to have more severe clinical symptomatology than the HC group, one-tailed analyses were used to assess between-group differences in clinical state, mood, emotion reactivity, and impulsivity.

To assess performance on the affective picture task, 2 × 4 mixed-model ANOVAs (two-tailed) were performed on response time and omission error data. For these analyses, we were interested in assessing the main effects of Diagnostic Group (HC and NSSI) and Affective Condition (Neutral, NSSI, Negative, and Positive) as well as the Group × Condition interaction. Additionally, 2 × 5 mixed-model ANOVAs (two-tailed) were performed in order to assess changes in clinical state as measured by the PANAS over the course of the task. For these ANOVAs, the repeated-measures factor Affective Condition included a baseline PANAS obtained before the task began. All mixed-model ANOVAs were subjected to Greenhouse-Geisser corrections when the assumption of sphericity was violated. Furthermore, when the omnibus, mixed-model ANOVAs indicated a significant main effect of Affective Condition and/or a Group × Condition interaction, *post hoc* repeated-measures ANOVAs were performed for each individual diagnostic group in order to assess changes in Affective Condition over time within each group; these *post hoc* assessments included Least Significant Difference (LSD) pairwise comparisons of each Affective Condition to baseline PANAS score.

### Imaging Methods

Imaging was performed on a Siemens Trio whole body 3T MRI scanner (Siemens Corporation, Erlangen, Germany) using a quadrature RF head coil; 40 contiguous coronal slices were acquired, providing whole brain coverage (5 mm, 0 mm skip). Images were collected using a single shot, gradient pulse echo sequence (TR=3,000 ms, TE=30 ms, flip angle=90, FOV=20 cm, 64 × 64 acquisition matrix, plane resolution 3.125 mm^3^ × 3.125 mm^3^ ×3.125 mm^3^); 50 images per slice were collected.

Functional MRI images were analyzed using Statistical Parametric Mapping (SPM8, version 4290, Wellcome Department of Imaging Neuroscience, University College, London, UK) software package running in Matlab (version R2010b, MathWorks, Natick, MA, USA). First, blood oxygen level dependent (BOLD) fMRI data were corrected for motion using a two-step, intra-run realignment algorithm, which used the mean image created after the first realignment as a reference (≥3 mm of translational or rotational motion was exclusionary, but no participants exceeded this movement threshold). Realigned images were then normalized in Montreal Neurological Institute (MNI) stereotactic space, resampled into 2 mm^3^ voxels, and spatially smoothed using an isotropic Gaussian kernel (8 mm full width at half maximum) without global scaling. High-pass temporal filtering (cutoff=128 s) was applied, and serial autocorrelations were modeled with SPM8's AR(1) model.

A first-level fixed-effect model was constructed for each participant in which image condition effects at each voxel were calculated using a t-statistic, producing a statistical image contrast for each of the four picture conditions (neutral, NSSI, negative, positive) with the fixation period subtracted. Movement parameters from the realignment stage were entered as covariates in order to control for participant movement. A general linear model (GLM) was conducted on the *t*-contrast images generated in the previous single-subject analyses. These second level analyses were conducted using a 2 (diagnostic group) × 4 (picture condition) factorial design. The GLM analyses were conducted using *a priori* region-of-interest (ROI) bilateral masks created using the Wake Forest University PickAtlas utility (45) for the amydala, CC, OFC, NAcc, and VTA. The statistical threshold was set at uncorrected *p ≤* 0.05 and a minimum cluster extent *k*≥5 contiguous voxels. Post hoc analyses with independent t-tests were performed within SPM in ROI clusters showing a significant diagnostic group × image condition interaction in each ANOVA. To control for multiple comparisons, we used a Bonferroni-corrected voxelwise threshold (*p* < 0.015) for these *post hoc* tests.

## Results

### Demographic and Clinical Variables

Detailed demographic and clinical information (including subscale data) for the HC and NSSI groups are available elsewhere [see (24)]. **Table 1** provides the means for the measures overall. The NSSI and HC groups were well matched for age, and although a trend emerged for the HC group to have slightly more years of education than the NSSI group (*p*=.063), IQ was not significantly different between the groups. With regard to NSSI exposure, the NSSI group reported an average of 6.00 years (*SD*=3.91) of engaging in NSSI behaviors, 124.09 (*SD*=118.74) lifetime NSSI episodes, and 1.07 (*SD*=1.59) NSSI episodes within the past week. Clinical state and mood assessments indicated that the NSSI group had greater severity of clinical symptomatology and mood disturbance relative to the HC group across all rating scales. Similarly, the NSSI group endorsed poorer emotion regulation on the WBSI and ERS compared to the HC group. Additionally, the NSSI group reported significantly higher levels of impulsivity on the UPPS relative to the HC group.

**Table 1:** Demographic and clinical characteristics of healthy control (HC) and nonsuicidal self-injury (NSSI) participants.

Variable	Control *n=15*	NSSI *n=15*	Analyses of Variance^a^	
	***M* ± *SD***	***M* ± *SD***	***F***	***p* (*η^2^*) 2-tailed**
Age	22.80 ± 3.28	21.27 ± 3.67	1.455	.238 (.*049*)
Education (yrs)	15.47 ± 2.30	13.73 ± 2.60	3.741	.063 (*.118*)
IQ (WASI)	116.80 ± 12.68	112.07 ± 12.46	1.063	.311 (*.037*)
**SITBI**	***M* ± *SD***	***M* ± *SD***	***95% CI***	
Age of 1^st^ NSSI	*–*	15.27 ± 1.98	[14.17, 16.36]	
Duration of NSSI (yrs)	*–*	6.00 ± 3.91	[3.84, 8.17]	
*NSSI Episodes*				
Lifetime^b^	*–*	124.09 ± 118.74	[44.32, 203.86]	
Past Year^b^	*–*	38.82 ± 54.04	[2.51, 75.12]	
Past Month^c^	*–*	5.00 ± 7.93	[0.42, 9.58]	
Past Week^c^	*–*	1.07 ± 1.59	[0.15, 1.99]	
**Clinical Measures**	***M* ± *SD***	***M* ± *SD***	***F***	***p* (*η^2^*) 1-tailed**
*STAI*				
State Anxiety	28.93 ± 7.10	43.40 ± 9.20	**23.272**	**<.001 (*.454*)**
Trait Anxiety	29.73 ± 5.84	55.60 ± 11.90	**57.105**	**<.001 (*.671*)**
*BDI*				
State Depression	1.07 ± 1.67	20.17 ± 12.63	**33.880**	**<.001 (*.575*)**
*MASQ*				
Total	92.53 ± 12.32	156.67 ± 39.90	**56.391**	**<.001 (.*668*)**
*PANAS (pre scan)*				
Positive Affect	30.47 ± 7.30	21.93 ± 4.88	**14.177**	**<.001 (.*336*)**
Negative Affect	10.20 ± 0.56	14.13 ± 5.15	**8.638**	**.004 (*.236*)**
*POMS* ^d^				
Total Mood Disturbance	−6.00 ± 10.63	61.07 ± 38.71	**41.766**	**<.001(*.607*)**
**Emotion Regulation**	***M* ± *SD***	***M* ± *SD***	***F***	***p* (*η^2^*) 1-tailed**
*WBSI*				
Total	30.07 ± 9.18	51.20 ± 14.16	**23.510**	**<.001 (.456)**
*ERS* ^d^				
Total	9.80 ± 6.37	23.72 ± 22.00	**21.625**	**<.001 (.445)**
**Impulsivity**	***M* ± *SD***	***M* ± *SD***	***F***	***p* (*η^2^*) 1-tailed**
*UPPS*				
Total	17.33 ± 5.22	26.53 ± 8.82	**12.099**	**.001 (*.302*)**

Significant Effects in bold.

a df=1,28 unless otherwise indicated.

b n=11

c n=14

d df=1,27

BDI, Beck Depression Inventory; ERS, Emotion Reactivity Scale; MASQ, Mood and Anxiety Symptoms Questionnaire; PANAS, Positive and Negative Affect Schedule; POMS, Profile of Mood States; SITBI, Self-Injurious Thoughts and Behaviors Interview; STAI, State-Trait Anxiety Inventory; UPPS, UPPS Impulsive Behavior Scale; WASI, Wechsler Abbreviated Scale of Intelligence; WBSI, White Bear Suppression Inventory.

### Response Times

Between-group comparisons examining performance on the affective picture task (**Table 2**), indicated that both the HC and NSSI groups had similar response times [*F*(1,27)=0.67, *p*=.42] and omission errors [*F*(1,27)=1.08, *p*=.31]. Additionally, repeated-measures comparisons indicated similar response times [*F*(1.34,36.14)=0.19, *p*=.73] and omission errors [*F*(3,81)=0.67, *p*=.57] across all affective conditions. There were no significant Group × Condition interactions for either response time [*F*(1.34,36.14)=0.17, *p*=.75] or omission errors [*F*(3,81)=1.34, *p*=.27].

**Table 2:** Affective picture task performance.

Variable	HC *n=15*	NSSI *n=15*
Performance	*M* ± *SD*	*M* ± *SD*
*Response Time (ms)*		
Neutral	695.74 ± 277.27	667.34 ± 494.82
NSSI	706.98 ± 204.33	631.04 ± 172.59
Negative	711.91 ± 244.24	620.38 ± 149.04
Positive	681.77 ± 251.99	611.89 ± 179.94
*Omission Errors*		
Neutral	0.00 ± 0.00	0.21 ± 0.58
NSSI	0.07 ± 0.26	0.14 ± 0.54
Negative	0.00 ± 0.00	0.14 ± 0.54
Positive	0.00 ± 0.00	0.14 ± 0.54

HC, healthy control; NSSI, nonsuicidal self-injury.

### Mood State Changes

**Figure 1:**
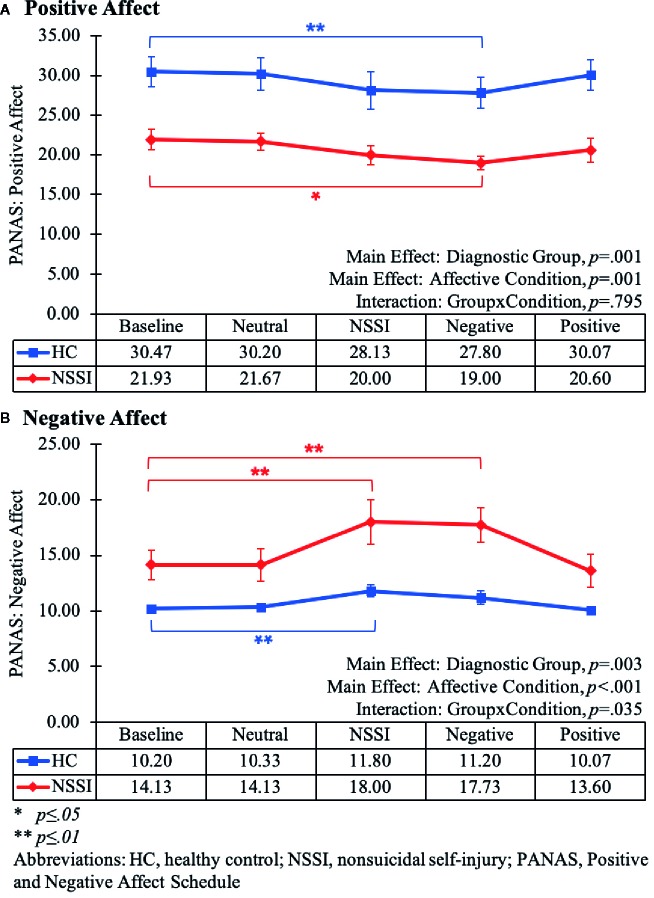
Line graphs illustrating the changes in positive **(A)** and negative affect **(B)** as measured by the Positive and Negative Affect Schedule (PANAS) during each Affective Condition of the Affective Picture Task (Baseline, Neutral, NSSI, Negative, and Positive). The main effect and interaction results from the mixed-model (2x5) ANOVAs are listed at the bottom of each graph. The results from the post hoc Least Significant Difference (LSD) pairwise comparisons of each Affective Condition relative to the Baseline Condition are noted within the graph (*p ≤ 0.05; **p ≤ 0.01).

Mixed-model ANOVAs assessing changes in PANAS scores across the different Affective Conditions indicated significant between-group differences with the HC group reporting higher overall positive affect [*F*(1,28)=15.14, *p* < .01] and lower overall negative affect [*F*(1,28)=10.80, *p* < .01] relative to the NSSI group (**Figure 1**). Additionally, as would be expected given that participants were viewing different types of images, mood state varied significantly across the affective conditions for ratings of positive [*F*(2.57,71.87)=7.20, *p* < .01] and negative mood [*F*(2.38,66.77)=13.26, *p* < .01]. Of note, there was a significant Group × Condition interaction for negative [*F*(2.38,66.77)=3.31, *p*=.04], but not for positive mood [*F*(2.57,71.87)=0.30, *p*=.80]. Whereas the groups reported similar changes in positive mood over time, the HC and NSSI groups reported different levels of negative mood across the affective conditions. Post hoc LSD pairwise comparisons indicated participants in both groups reported significant decreases in positive affect after viewing negative images (**Figure 1A**) and significant increases in negative affect after viewing NSSI images (**Figure 1B**) relative to baseline. However, the NSSI group had significantly higher negative mood ratings after viewing negative images relative to pretask baseline; no such changes were noted in the HC group.

### Functional Neuroimaging

Both the HC and NSSI groups demonstrated significant activation during the affective picture task within ROIs associated with emotion regulation (amygdala, CC, and OFC); however, neither group demonstrated significant activation within ROIs associated with reward circuitry (NAcc and VTA) during any of the affective conditions.

**Figure 2:**
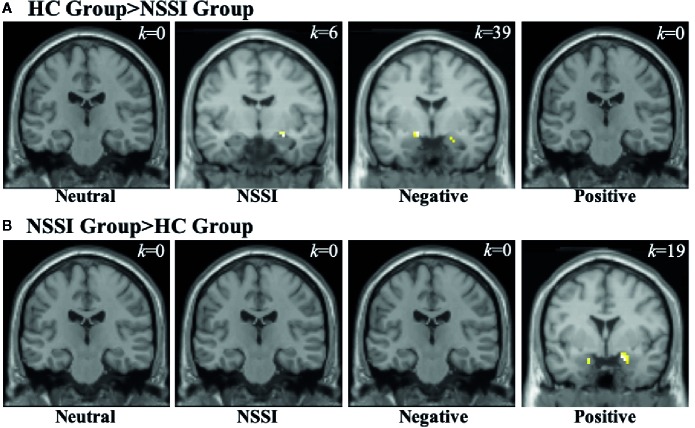
Functional MRI images demonstrating between-groups differences in activation within the amygdala region of interest during the Neutral, NSSI, Negative, and Positive Conditions of the Affective Picture Task. The two between-group comparisons are the healthy control (HC) greater than nonsuicidal self injury (NSSI) contrast **(A)** and the NSSI greater than HC contrast **(B)**. The slice images presented are from the coordinates of the most significant activation cluster for each contrast. The significant threshold was set at uncorrected p ≤ 0.05 and a minimum cluster extent k ≥ 5 contiguous voxels.

Within the amygdala ROI (**Table 3**), overall group averages indicated that the HC group demonstrated significant activation while viewing neutral (*k*=70), NSSI (*k*=47), negative (*k*=90) and positive (*k*=5) images. The NSSI group demonstrated significant amygdalar activation while viewing NSSI (*k*=40), negative (*k*=7), and positive (*k*=19), images; however, no significant amygdalar activation was detected while viewing neutral images. Post hoc between group comparisons (**Figure 2**; for glass brain images see **Supplemental Figure 1**) indicated that the HC group had significantly greater right amygdalar activation while viewing NSSI images (*k*=6) and greater bilateral amygdalar activation while viewing negative images (*k*=39) relative to the NSSI group. Conversely, the NSSI group had significantly greater bilateral amygdalar activation while viewing *positive* images (*k*=19) relative to the HC group. The HC and NSSI groups did not significantly differ on amygdalar activation while viewing neutral images.

**Table 3:** Affective picture task local maxima fMRI activation: Amygdala region of interest.

ROI	Contrast	Coordinate label	Cluster size k (Voxels)	MNI Coordinates			*t* Score	p*
				x	Y	Z		
**Amygdala**								
*Neutral Images*								
Group Averages	HC Group	Left Amygdala	47	−27	−3	−27	3.80	< .001
		Right Amygdala	23	21	−6	−15	2.93	.002
	NSSI Group	None	–	–	–	–	–	–
Group Comparison	HC > NSSI	None	–	–	–	–	–	–
	NSSI > HC	None	–	–	–	–	–	–
*NSSI Images*								
Group Averages	HC Group	Left Amygdala	42	−18	−3	−12	3.10	.001
		Right Amygdala	5	24	−9	−15	2.60	.005
	NSSI Group	Left Amygdala	16	−18	−3	−15	2.59	.005
		Left Amygdala	9	−30	0	−27	2.41	.009
		Right Amygdala	15	33	−3	−24	2.39	.009
Group Comparison	HC > NSSI	Right Amygdala	6	27	−9	−15	2.18	.016
	NSSI > HC	None	–	–	–	–	–	–
*Negative Images*								
Group Averages	HC Group	Left Amygdala	41	−15	−3	−15	4.03	< .001
		Right Amygdala	49	24	0	−21	3.54	< .001
	NSSI Group	Left Amygdala	7	−21	−9	−15	2.50	.007
Group Comparison	HC > NSSI	Left Amygdala	13	−15	−3	−12	3.15	.001
		Right Amygdala	26	24	3	−21	3.15	.001
	NSSI > HC	None	–	–	–	–	–	–
*Positive Images*								
Group Averages	HC Group	Left Amygdala	5	−24	−3	−27	2.85	.003
	NSSI Group	Right Amygdala	12	18	0	−18	2.95	.002
		Left Amygdala	7	−18	0	−24	2.68	.004
Group Comparison	HC > NSSI	None	–	–	–	–	–	–
	NSSI > HC	Right Amygdala	14	21	0	−21	3.14	.001
		Left Amygdala	5	−18	0	−21	2.51	.007

*p≤.05, k≥5.

BA, Brodmann Area; fMRI, functional magnetic resonance imaging; HC, healthy control; MNI, Montreal Neurological Institute; NSSI, nonsuicidal self-injury ROI, region of interest.

**Figure 3:**
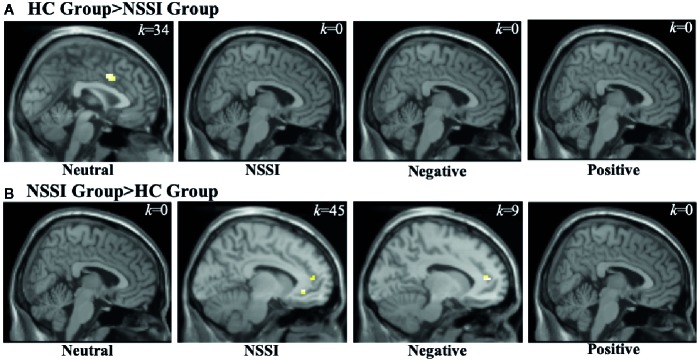
Functional MRI images demonstrating between-group differences in activation within the cingulate cortex (CC) region of interest during the Neutral, NSSI, Negative, and Positive Conditions of the Affective Picture Task. The two between-group comparisons are the healthy control (HC) greater than nonsuicidal self injury (NSSI) contrast **(A)** and the NSSI greater than HC contrast **(B)**. The slice images presented are from the coordinates of the most significant activation cluster for each contrast. The significant threshold was set at uncorrected p ≤ 0.05 and a minimum cluster extent k ≥ 5 contiguous voxels.

Within the CC ROI (**Table 4**), overall group averages indicated that the HC group demonstrated significant activation while viewing neutral (*k*=121) and NSSI (*k*=34) images but not while viewing negative or positive images. The NSSI group demonstrated significant CC activation while viewing neutral (*k*=9), NSSI (*k*=111) negative (*k*=28), and positive (*k*=12) images. Post hoc between group comparisons (**Figure 3**; for glass brain images see **Supplemental Figure 2**) indicated that the HC group had significantly greater right dorsal anterior CC activation while viewing neutral images (*k*=34) relative to the NSSI group. Conversely, the NSSI group had significantly greater bilateral CC activation while viewing NSSI images (*k*=45) and greater left dorsal anterior CC activation while viewing negative images (*k*=9) relative to the HC group. No differences were detected between the HC and NSSI groups while viewing positive images.

**Table 4:** Affective picture task local maxima fMRI activation: Cingulate cortex region of interest.

ROI	Contrast	Coordinate label	Cluster size k (Voxels)	MNI Coordinates				*t* Score	p*	
				x	Y		Z			
**Cingulate Cortex (CC)**										
*Neutral Images*										
Group Averages	HC Group	Right Frontal Cortex BA8	111	6	12		42	2.90	.002	
		Left Dorsal Posterior CC BA31	5	−9	−36		51	2.15	.017	
		Center Dorsal Anterior CC BA32	5	0	27		27	1.93	.028	
	NSSI Group	Left Dorsal Anterior CC BA32	9	−6	33		−6	2.02	.023	
Group Comparison	HC > NSSI	Right Dorsal ACC BA32	34	0	9		36	2.19	.015	
	NSSI > HC	None	–	–	–		–	–	–	
*NSSI Images*										
Group Averages	HC Group	Center Anterior CC BA24	34	0	3		33	2.47	.008	
	NSSI Group	Left Dorsal Anterior CC BA32	26	−9	33		−9	4.12	< .001	
		Center Anterior CC BA24	73	0	3		30	3.06	.001	
		Left Dorsal Anterior CC BA32	12	−3	51		9	2.48	.007	
Group Comparison	HC > NSSI	None	–	–	–		–	–	–	
	NSSI > HC	Left Dorsal ACC BA32	12	−9	36		−6	2.72	.004	
		Right Frontal Cortex BA8	19	9	33		27	2.39	.009	
		Left Dorsal ACC BA32	14	−3	51		9	2.30	.012	
*Negative Images*										
Group Averages	HC Group	None	–	–	–		–	–	–	
	NSSI Group	Right Dorsal Anterior CC BA32	15	3	36		−6	2.11	.019	
		Left Dorsal Anterior CC BA32	13	−3	48		9	2.01	.023	
Group Comparison	HC > NSSI	None	–	–	–		–	–	–	
	NSSI > HC	Left Dorsal ACC BA32	9	−12	45		9	2.42	.008	
*Positive Images*										
Group Averages	HC Group	None	–	–	–		–	–	–	
	NSSI Group	Left Dorsal Anterior CC BA32	12	−9	33		−9	2.70	.004	
Group Comparison	HC > NSSI	None	–	–	–		–	–	–	
	NSSI > HC	None	–	–	–		–	–	–	

*p≤.05, k≥5.

BA, Brodmann Area; CC, anterior cingulate cortex; fMRI, functional magnetic resonance imaging; HC, healthy control; MNI, Montreal Neurological Institute; NSSI, nonsuicidal self-injury; ROI, region of interest.

**Figure 4:**
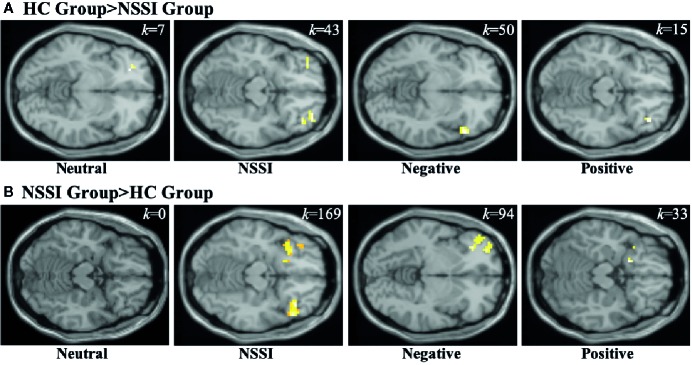
Functional MRI images demonstrating between-group differences in activation within the orbitofrontal cortex (OFC) region of interest during the Neutral, NSSI, Negative, and Positive Conditions of the Affective Picture Task. The two between-group comparisons are the healthy control (HC) greater than nonsuicidal self- injury (NSSI) contrast **(A)** and the NSSI greater than HC contrast **(B)**. The slice images presented are from the coordinates of the most significant activation cluster for each contrast. The significant threshold was set at uncorrected p ≤ 0.05 and a minimum cluster extent k ≥ 5 contiguous voxels.

Within the OFC ROI (**Table 5**), overall group averages indicated that both the HC and NSSI groups demonstrated significant activation while viewing all images but differed with regard to the magnitude of activation per condition: OFC activation for the HC group during neutral (*k*=90), NSSI (*k*=240), negative (*k*=307) and positive (*k*=21) images contrasted with the activation for the NSSI group during neutral (*k*=42), NSSI (*k*=458), negative (*k*=498) and positive (*k*=205) images. Post hoc between group comparisons (**Figure 4**; for glass brain images **Supplemental Figure 3**) indicated that the HC group had significantly greater left ventral OFC activation for neutral images (*k*=7), bilateral OFC and right anterior prefrontal cortex activation (*k*=43) for NSSI images, bilateral ventral OFC activation for negative images (*k*=50), and right ventral OFC activation for positive images (*k*=15) relative to the NSSI group. The NSSI group had significantly greater bilateral OFC and right anterior prefrontal cortex activation for NSSI images (*k*=169), bilateral OFC and left prefrontal cortex activation for negative images (*k*=94), and bilateral ventral OFC and right anterior prefrontal cortex activation for positive images (*k*=33) relative to the HC group.

**Table 5:** Affective picture task local maxima fMRI activation: Orbitofrontal cortex region of interest.

ROI	Contrast	Coordinate label	Cluster size k (Voxels)	MNI Coordinates				*t* Score	p*	
				x	Y		Z			
**Orbitofrontal Cortex (OFC)**										
*Neutral Images*										
Group Averages	HC Group	Right Ventral OFC BA47	13	48	18		−9	2.66	.004	
		Right Ventral OFC BA47	29	33	33		−21	2.44	.008	
		Left Ventral OFC BA47	41	−48	15		−6	2.34	.011	
		Right Insula BA13	7	33	21		−9	2.32	.011	
	NSSI Group	Right Medial Ventral OFC BA11	11	24	36		−18	2.83	.003	
		Left Medial Ventral OFC BA11	6	−12	45		−15	2.59	.006	
		Right Ventral OFC BA47	11	48	42		−12	2.18	.016	
		Left Medial Ventral OFC BA11	6	−21	15		−15	2.06	.021	
		Left Ventral OFC BA47	8	−33	30		−6	2.03	.022	
Group Comparison	HC > NSSI	Left Ventral OFC BA47	7	−27	39		−9	2.24	.013	
	NSSI > HC	None	–	–	–		–	–	–	
*NSSI Images*										
Group Averages	HC Group	Left Medial Ventral OFC BA11	90	−24	36		−21	3.88	< .001	
		Right Medial Ventral OFC BA11	55	24	36		−18	3.64	< .001	
		Right Ventral OFC BA47	42	39	42		−12	3.48	< .001	
		Left Anterior PFC BA10	26	−33	60		−3	3.46	< .001	
		Right Ventral OFC BA47	16	51	18		−12	3.33	.001	
		Left Ventral OFC BA47	11	−54	33		−9	2.80	.003	
	NSSI Group	Right Medial Ventral OFC BA11	216	24	33		−18	4.26	< .001	
		Left Medial Ventral OFC BA11	217	−24	27		−18	3.95	< .001	
		Left Anterior PFC BA10	14	−33	60		−3	3.11	.001	
		Left Anterior PFC BA10	6	−45	51		−6	3.04	.006	
		Right Anterior PFC BA10	5	27	63		−3	2.04	.022	
Group Comparison	HC > NSSI	Right Anterior PFC BA10	19	36	51		−15	2.64	.005	
		Right Ventral OFC BA47	12	39	42		−12	2.53	.006	
		Left Ventral OFC BA47	12	−33	45		−18	2.49	.007	
	NSSI > HC	Right Ventral OFC BA47	83	39	30		−15	3.37	.001	
		Left Ventral OFC BA47	58	−30	21		−12	2.86	.003	
		Right Anterior PFC BA10	11	36	57		−3	2.50	.007	
		Left Ventral OFC BA47	12	−39	39		−18	2.39	.009	
		Left Medial Ventral OFC BA11	5	−18	15		−15	2.32	.011	
*Negative Images*										
Group Averages	HC Group	Right Anterior PFC BA10	195	30	48		−18	3.48	< .001	
		Left Medial Ventral OFC BA11	67	−24	36		−21	3.16	.001	
		Left Ventral OFC BA47	45	−54	24		−6	2.47	.008	
	NSSI Group	Left Ventral OFC BA47	116	30	30		−21	3.46	< .001	
		Right Ventral OFC BA47	59	42	42		−18	3.26	.001	
		Left Ventral OFC BA47	307	−27	15		−24	3.25	.001	
		Right Ventral OFC BA47	16	54	36		−6	3.22	.001	
Group Comparison	HC > NSSI	Right Ventral OFC BA47	24	54	24		−9	2.82	.003	
		Right Ventral OFC BA47	20	36	18		−21	2.45	.008	
		Left Ventral OFC BA47	6	−39	24		−12	1.88	.031	
	NSSI > HC	Left Ventral OFC BA47	19	−36	33		−6	2.60	.005	
		Left Anterior PFC BA10	47	−33	51		−6	2.36	.010	
		Right Ventral OFC BA47	17	36	45		−15	2.13	.018	
		Left Ventral OFC BA47	11	−33	24		−18	2.10	.019	
*Positive Images*										
Group Averages	HC Group	Left Ventral OFC BA47	5	−36	30		−21	2.12	.018	
		Right Ventral OFC BA47	10	36	33		−18	2.07	.020	
		Left Medial Ventral OFC BA11	6	−24	36		−21	2.02	.023	
	NSSI Group	Left Ventral OFC BA47	164	−27	30		−24	4.09	< .001	
		Right Medial Ventral OFC BA11	28	24	36		−18	2.62	.005	
		Right Ventral OFC BA47	13	45	30		−12	2.21	.014	
Group Comparison	HC > NSSI	Right Ventral OFC BA47	15	39	39		−15	2.28	.012	
	NSSI > HC	Left Medial Ventral OFC BA11	9	−18	12		−18	2.43	.008	
		Right Ventral OFC BA47	7	48	33		−15	1.97	.025	
		Left Ventral OFC BA47	12	−42	18		−15	1.95	.027	
		Right Anterior PFC BA10	5	39	57		−3	1.87	.031	

*p≤.05, k≥5.

BA, Brodmann Area; fMRI, functional magnetic resonance imaging; HC, healthy control; MNI, Montreal Neurological Institute; NSSI, nonsuicidal self-injury; OFC, orbitofrontal cortex; PFC, prefrontal cortex; ROI, region of interest.

The GLM analyses described above were conducted using *a priori* ROI masks with the significance threshold set at uncorrected *p ≤* 0.05 and a minimum cluster extent *k*≥5 contiguous voxels; *post hoc* independent *t*-tests were performed in SPM using Bonferroni-corrected voxel-wise threshold of *p* < 0.015. We also repeated all fMRI analyses using a moderate significance threshold (*p ≤* 0.01 and a minimum cluster extent *k*≥10 contiguous voxels) and conservative significance threshold (*p ≤* 0.001 and a minimum cluster extent *k*≥10 contiguous voxels). Analyses using the moderate significance threshold demonstrated increased amygdala activation in the HC group (*k*=13) relative to the NSSI group (*k*=0) when viewing negative images (**Supplemental Table 1**); there were no significant differences between the groups in the CC (**Supplemental Table 2**) or the OFC (**Supplemental Table 3**) ROIs. Analyses using the conservative significance threshold did not reveal any significant activation in any of the ROIs suggesting that this threshold may be too conservative for a pilot study with a reduced sample size.

## Discussion

In this pilot investigation, individuals who engage in NSSI behavior exhibited altered activation of emotion processing and regulation circuitry when viewing affective images relative to HC participants. Specifically, the HC group exhibited greater amygdalar activation in response to NSSI and negative images compared to the NSSI group, who demonstrated greater amygdalar activation in response to positive images. Interestingly, an opposite pattern of brain activation was observed within the CC ROI, with the NSSI group exhibiting greater activation during NSSI and negative images relative to the HC group. Additionally, within the OFC ROI, both the HC and NSSI groups demonstrated increased activation during NSSI, negative, and positive images; however, the NSSI group always exhibited a greater magnitude of activation differences relative to the HC group. These results suggest that individuals who engage in NSSI behaviors utilize different areas of emotion regulation circuitry relative to HCs, with decreased amygdalar and increased CC and OFC activation during the processing of more aversive stimuli (NSSI and negative images) and increased amygdalar and OFC activation during positive stimuli.

Neither the NSSI nor the HC group demonstrated significant activation within regions typically associated with reward during any of the affective conditions. However, the IAPS images used in the affective picture task were specifically selected because they had average normative arousal ratings. For this reason, even the positive images may not have provided enough of a reward to sufficiently activate the NAcc and VTA ROIs within the reward circuitry. It was also the case that ratings of positive affect collected throughout the affective picture task remained primarily stable across the affective conditions relative to baseline. One exception to this was the significant decrease in positive affect observed in both the HC and NSSI groups after participants viewed negative images.

Importantly, behavioral data and clinical ratings collected during the task indicated that all participants were actively engaged in the task. Ratings of positive and negative affect changed throughout the course of the task in concordance with the type of affective stimuli presented. The NSSI group did report lower positive and higher negative affect than the HC group overall as well as greater increased negative affect after the NSSI and negative image affective conditions. However, this was expected given the higher levels of clinical symptoms reported by the NSSI group.

Although other explanations are possible, the lower amygdalar activation in response to NSSI images in the NSSI group participants is consistent with the idea of diminished aversion. On average, the NSSI participants had been engaging in NSSI behaviors for 6 years and had high levels of lifetime NSSI episodes as well as recent engagement (past month, past week) in NSSI behaviors. In contrast, our HC participants with no past history of self-injurious behavior responded to the NSSI images with significantly greater amygdalar activation. It also warrants mention that both groups of participants experienced a significant increase in negative affect while viewing the NSSI images. These findings suggest that, consistent with Allen and Hooley (18), NSSI images are experienced in a negative way; they are not explicitly interpreted as positive stimuli. However, to the extent that amygdala activation can be viewed as an indicator of threat or salience, our finding suggests, at the neurobiological level, that NSSI images are less emotionally aversive to people with NSSI histories. This may be because such individuals are more habituated to images of scalpels, razors, or cut wrists. Alternatively, the emotional relief that results from NSSI may, *via* a conditioning process, have changed the “meaning” or salience of NSSI stimuli in important ways, and the increased CC and OFC activity in NSSI participants during exposure to NSSI images may reflect this. Further, the similar amygdalar response of the NSSI participants to negative images as well as to NSSI images may also be indicative of a diminished aversion to unpleasant stimuli more broadly. It is possible, however, that since the negative affective condition images were always viewed after the NSSI images, some priming or carry over effects from the NSSI stimuli may have been present. Future studies would do well to vary to order of presentation of the affective stimuli to investigate these possibilities. The order of presentation of the different conditions in the current study was designed with human subjects' concerns in mind. Presenting positive images in the final block allowed participants to leave the study in a more positive and less negative mood than might otherwise be the case.

Despite the mood benefits that result from self-injurious behaviors and from pain, we found little evidence of increased activation in reward processing areas when people who engage in NSSI view NSSI images. Positive mood did not increase and no significant activation was detected within the NAcc or VTA in either participant group. We did, however, find increased activation in the OFC within the NSSI participants in the context of viewing NSSI images. To the extent that the OFC is implicated in coding reward representations (46, 47) this finding supports the idea that NSSI stimuli may have a different and perhaps more nuanced meaning for people who engage in NSSI versus those who do not. More research is needed to further explore this issue in those who engage in NSSI.

Although intriguing, study findings must be considered in light of several limitations. The current study was a pilot investigation with a relatively modest sample size (*N*=30). Although a modest sample size is not unusual for preliminary studies of this type, we were aware of the resulting loss of statistical power. We therefore adopted an ROI approach to reduce the number of statistical tests and decrease the likelihood of Type 1 error. In parallel with this we utilized more liberal significance thresholds in our fMRI analyses to decrease the likelihood of Type II errors. When we repeated the fMRI analyses using a moderate significance threshold there was evidence of decreased amygdala activation in the NSSI group relative to the HC group when viewing negative images. There were no group differences in any of the ROIs when a conservative significance threshold was used. This threshold may be too conservative for a pilot study with a reduced sample size.

Given the modest sample size of this pilot investigation, it is important to emphasize the exploratory nature of the findings and underscore the need for replication in a larger sample. Conservative statistical thresholds of *p*≤.001 reduce the likelihood of Type I errors and lower false discovery rates [e.g., (48, 49)]. Yet utilizing more conservative thresholds when the sample size is small greatly increases the likelihood of Type II errors. Power analyses of the current results (*p ≤* 0.05; *k*≥5) indicated that we would need to at least double our current sample size to a minimum of 30 participants per group (*N*≥60) in order to observe the same effect sizes at the more conservative statistical threshold (*p ≤* 0.001; *k*≥10). In fact, some researchers have suggested that fMRI studies with fewer than 50 participants per group have limited statistical power [e.g., (50)]. Sample sizes of that magnitude are clearly not pilot or exploratory investigations, and with the considerable expense of neuroimaging, are cost prohibitive for most researchers. This preliminary work suggests that examining how people who engage in NSSI process NSSI images may be a productive avenue of inquiry for future research efforts.

Additionally, the current study focused on NSSI imagery involving skin-cutting, which is more prevalent in females (32), and in order to maximize homogeneity of our sample, only adult females were recruited. The majority also had comorbid BPD and depression as well as other disorders in several cases. Therefore, our results may not generalize to other forms of NSSI, mixed-sex samples, or individuals without comorbid diagnoses. Given that Plener et al. (22) have reported amygdala hyperactivation to emotional images in a small sample of adolescent females with NSSI (compared to adolescent females with no history of NSSI) it will also be important to examine how variables such as age and years of NSSI engagement play a role. Poon and colleagues (31) have suggested that repeated engagement in NSSI may alter reward circuitry and dampen emotional sensitivity and reactivity. To the extent that this is the case, careful attention to the characteristics of the sample being studied is of considerable importance. A strength of the current study is that our NSSI group was well-characterized with an extensive history of NSSI behavior (*M*=6.00 years) as well as acute symptomatology (*M*=1.07 NSSI episodes in the past week). However, future studies should examine NSSI behavior longitudinally from first NSSI episode, documenting how these behaviors develop and change over time.

Although preliminary, the finding of decreased amygdalar activation to NSSI images in people with significant NSSI engagement relative to HCs with no NSSI history is suggestive of a diminished aversion to NSSI stimuli that is more implicit than explicit. Implicit aversion to NSSI images has been demonstrated in behavioral studies (14) but not, to date, in a neuroimaging study. Importantly, interventions designed to reduce NSSI by re-establishing aversion to NSSI stimuli are now being developed. Franklin et al. (51) have developed an engaging, game-like mobile App that utilizes a form of Pavlovian conditioning to treat NSSI. In the course of a 1- to 2-minute game, participants have to correctly pair a stimulus picture with its “match.” Importantly, images of cutting are always matched with aversive pictures (e.g., snakes, toenail fungus, etc.). Results from three randomized controlled trials provide support for this approach, highlighting the potential value of intervention efforts designed to increase aversion to NSSI stimuli. Whether amygdala activation to NSSI images might be eventually be used in this context as potential neurobiological marker of treatment success or relapse potential is an intriguing possibility, and underscores the importance of additional research in this area.

## Data Availability Statement

The datasets generated for this study are available on request to the corresponding author.

## Ethics Statement

The studies involving human participants were reviewed and approved by (1) Harvard University Committee on the Use of Human Subjects and (2) The McLean Hospital Institutional Review Board. The patients/participants provided their written informed consent to participate in this study.

## Author Contributions

JH and SG designed the study and developed the study protocol with assistance from SB and MD. JH, MD, and SB collected the clinical and demographic data. Neuroimaging data were primarily acquired by MD with additional assistance from JH and SB. AG performed the neuroimaging analyses and MD conducted additional statistical analyses and interpreted the data under the supervision of JH and SG. JH, MD, and SG wrote the manuscript. All authors contributed revisions and read and approved the submitted version.

## Funding

Financial support for this research was provided by a grant from the Harvard University William F. Milton Fund awarded to JH.

## Conflict of Interest

The authors declare that the research was conducted in the absence of any commercial or financial relationships that could be construed as a potential conflict of interest.
