# Supplementary material for: Decreased Amygdalar Activation to NSSI-Stimuli in People Who Engage in NSSI: A Neuroimaging Pilot Study
Source: Front Psychiatry. 2020 Apr 2;11:238. doi: 10.3389/fpsyt.2020.00238 (PMC7143895; doi:10.3389/fpsyt.2020.00238)
Supplement: Supplementary file 1 [file Presentation_1.pdf]

Supplemental Table 1. Affective Picture Task Local Maxima fMRI Activation: Amygdala Region of Interest

ROI	Contrast	Coordinate Label	Cluster Size k (Voxels)	MNI Coordinates x	y	z	*t* Score	*p**
**Amygdala**								
Neutral Images								
Group Averages	HC Group	Left Amygdala	10	-27	-3	-27	3.70	<.001
	NSSI Group	None	-	-	-	-	-	-
Group Comparison	HC>NSSI	None	-	-	-	-	-	-
	NSSI>HC	None	-	-	-	-	-	-
NSSI Images								
Group Averages	HC Group	None	-	-	-	-	-	-
	NSSI Group	None	-	-	-	-	-	-
Group Comparison	HC>NSSI	None	-	-	-	-	-	-
	NSSI>HC	None	-	-	-	-	-	-
Negative Images								
Group Averages	HC Group	Left Amygdala	22	-15	-3	-12	4.09	<.001
		Right Amygdala	26	24	0	-21	3.45	<.001
	NSSI Group	None	-	-	-	-	-	-
Group Comparison	HC>NSSI	Right Amygdala	13	24	3	-21	3.38	.001
	NSSI>HC	None	-	-	-	-	-	-
Positive Images								
Group Averages	HC Group	None	-	-	-	-	-	-
	NSSI Group	None	-	-	-	-	-	-
Group Comparison	HC>NSSI	None	-	-	-	-	-	-
	NSSI>HC	None	-	-	-	-	-	-

*$p \leq .01$, $k \geq 10$
Abbreviations: BA, Brodmann Area; fMRI, functional magnetic resonance imaging; HC, healthy control; MNI, Montreal Neurological Institute; NSSI, nonsuicidal self-injury ROI, region of interest

Supplemental Table 2. Affective Picture Task Local Maxima fMRI Activation: Cingulate Cortex Region of Interest

ROI	Contrast	Coordinate Label	Cluster Size k (Voxels)	MNI Coordinates x	y	z	*t* Score	*p**
**Cingulate Cortex (CC)**								
Neutral Images								
Group Averages	HC Group	Right Frontal Cortex BA8	21	6	12	42	3.03	.002
	NSSI Group	None	-	-	-	-	-	-
Group Comparison	HC>NSSI	None	-	-	-	-	-	-
	NSSI>HC	None	-	-	-	-	-	-
NSSI Images								
Group Averages	HC Group	None	-	-	-	-	-	-
	NSSI Group	Left Dorsal Anterior CC BA32	21	-9	33	-9	3.90	<.001
Group Comparison	HC>NSSI	None	-	-	-	-	-	-
	NSSI>HC	None	-	-	-	-	-	-
Negative Images								
Group Averages	HC Group	None	-	-	-	-	-	-
	NSSI Group	None	-	-	-	-	-	-
Group Comparison	HC>NSSI	None	-	-	-	-	-	-
	NSSI>HC	None	-	-	-	-	-	-
Positive Images								
Group Averages	HC Group	None	-	-	-	-	-	-
	NSSI Group	None	-	-	-	-	-	-
Group Comparison	HC>NSSI	None	-	-	-	-	-	-
	NSSI>HC	None	-	-	-	-	-	-

*p≤.01, k≥10

Abbreviations: BA, Brodmann Area; CC, anterior cingulate cortex; fMRI, functional magnetic resonance imaging; HC, healthy control; MNI, Montreal Neurological Institute; NSSI, nonsuicidal self-injury; ROI, region of interest

Supplemental Table 3. Affective Picture Task Local Maxima fMRI Activation: Orbitofrontal Cortex Region of Interest

ROI	Contrast	Coordinate Label	Cluster Size k (Voxels)	MNI Coordinates x	y	z	*t* Score	*p**
**Orbitofrontal Cortex (OFC)**								
Neutral Images								
Group Averages	HC Group	None	-	-	-	-	-	-
	NSSI Group	None	-	-	-	-	-	-
Group Comparison	HC>NSSI	None	-	-	-	-	-	-
	NSSI>HC	None	-	-	-	-	-	-
NSSI Images								
Group Averages	HC Group	Left Medial Ventral OFC BA11	28	-24	36	-21	3.34	.001
		Right Medial Ventral OFC BA11	13	24	33	-15	2.94	.002
	NSSI Group	Right Ventral OFC BA47	102	51	24	-15	3.88	<.001
		Left Ventral OFC BA47	65	-27	12	-27	3.66	<.001
Group Comparison	HC>NSSI	None	-	-	-	-	-	-
	NSSI>HC	None	-	-	-	-	-	-
Negative Images								
Group Averages	HC Group	Right Ventral OFC BA47	11	51	18	-12	2.76	.003
	NSSI Group	Left Ventral OFC BA47	17	-27	15	-24	3.18	.001
		Right Ventral OFC BA47	28	42	42	-18	3.10	.001
		Right Ventral OFC BA47	11	30	30	-21	3.09	.001
		Left Ventral OFC BA47	30	-45	42	-18	3.02	.002
		Left Ventral OFC BA47	25	-30	27	-24	3.01	.002
		Left Anterior PFC BA10	12	-48	48	-6	2.78	.003
Group Comparison	HC>NSSI	None	-	-	-	-	-	-
	NSSI>HC	None	-	-	-	-	-	-
Positive Images								
Group Averages	HC Group	None	-	-	-	-	-	-

	NSSI Group	None	-	-	-	-	-	-
Group Comparison	HC>NSSI	None	-	-	-	-	-	-
	NSSI>HC	None	-	-	-	-	-	-

*$p \leq .01$, $k \geq 10$

Abbreviations: BA, Brodmann Area; fMRI, functional magnetic resonance imaging; HC, healthy control; MNI, Montreal Neurological Institute; NSSI, nonsuicidal self-injury; OFC, orbitofrontal cortex; PFC, prefrontal cortex; ROI, region of interest

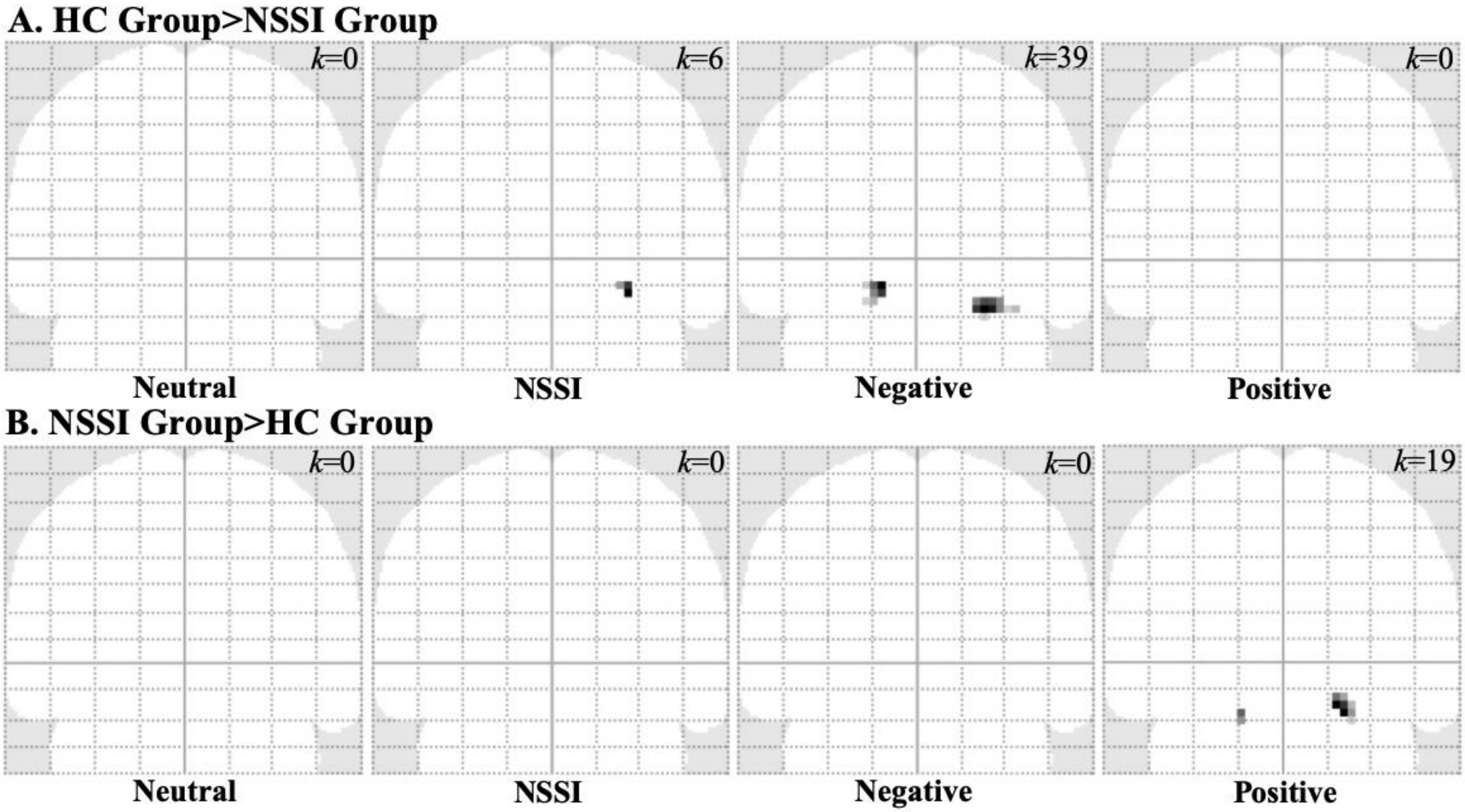

Supplemental Figure 1. Affective Picture Task fMRI Activation Healthy Control (HC) vs Nonsuicidal Self-Injury (NSSI) Glass Brain Image Group Comparisons: Amygdala Region of Interest.

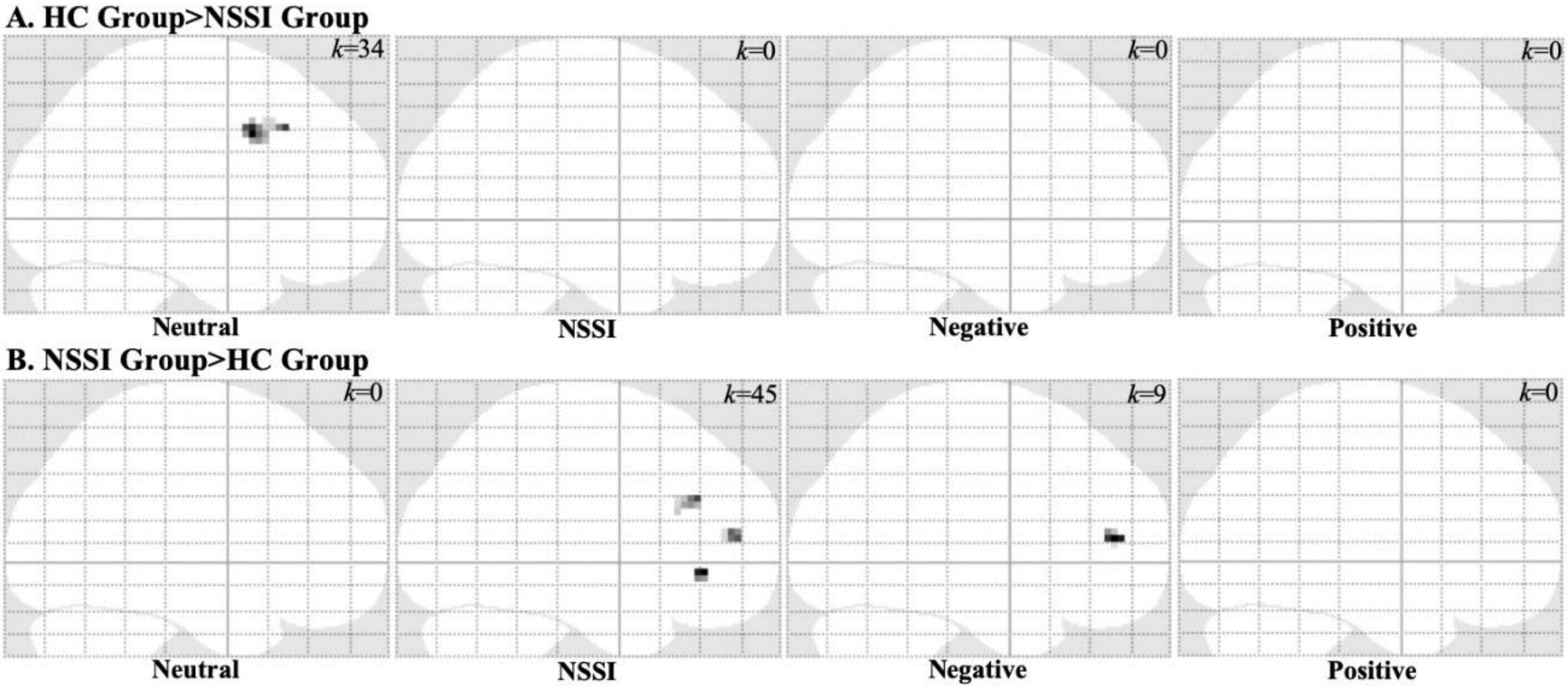

Supplemental Figure 2. Affective Picture Task fMRI Activation Healthy Control (HC) vs Nonsuicidal Self-Injury (NSSI) Glass Brain Image Group Comparisons: Cingulate Cortex Region of Interest.

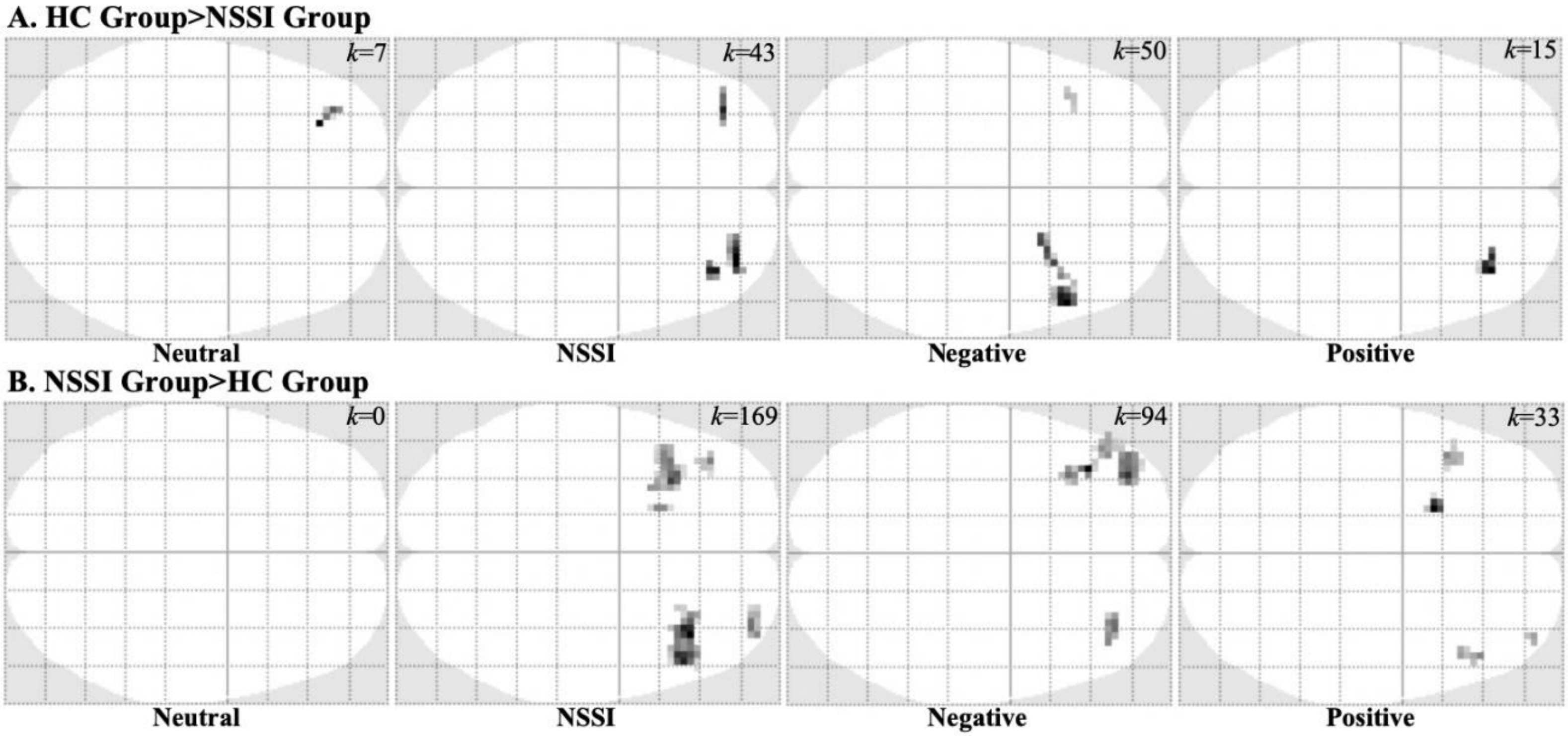

Supplemental Figure 3. Affective Picture Task fMRI Activation Healthy Control (HC) vs Nonsuicidal Self-Injury (NSSI) Glass Brain Image Group Comparisons: Orbitofrontal Cortex Region of Interest.
